# In Vitro Investigation of Bioactive Glass-Ceramic Composites Based on Biogenic Hydroxyapatite or Synthetic Calcium Phosphates

**DOI:** 10.1186/s11671-017-1895-1

**Published:** 2017-02-10

**Authors:** Nataliia Pinchuk, Oleksandr Parkhomey, Olena Sych

**Affiliations:** Frantsevich Institute for Problems of Materials Science of NAS of Ukraine, 3, Krzhyzhanovsky Str, Kyiv, 03680 Ukraine

**Keywords:** Hydroxyapatite, Calcium phosphate, Glass, Composite, Porosity, In vitro, Solubility

## Abstract

This in vitro investigation of the behavior of two types of calcium phosphate glass ceramics on the basis of phosphates of biogenic or synthetic origin prepared from initial mixtures with different particle size has revealed that some different factors affect the behavior, namely the phase composition of composite, fraction of open porosity, and average diameter of pore channels. It was established that the solubility of the composites on the basis of synthetic calcium phosphates and glass after 2 and 7 days contact with saline composites is the highest among the materials under study. First of all, this fact is related to the peculiarities of their phase composition, high fraction of open porosity, and high permeability. As for biogenic hydroxyapatite/glass materials, their solubility is several times lower in spite of close total porosity. The particle size of initial mixture practically does not affect the material solubility; the latter is only slightly lower for smaller particles.

## Background

Nowadays, regenerative medicine is one of the most developing trends in current science. One of its trends is the development of bioactive materials for replacement of defective areas of bone tissue [1–23]. Hydroxyapatite (HA), (Ca_10_(PO_4_)_6_(OH)_2_), is an analog of mineral component of human bone tissue. As it is HA that possesses high biocompatibility with human organism, many of the current biomaterial substitutes for bone tissue are various composites on the basis of HA and/or other calcium phosphate materials such as β-tricalciumphosphate (β-TCP), β-Ca_3_(PO4)_2_, calcium pyrophosphate (Ca_2_P_2_O_7_), tetracalciumphosphate (TTCP), and Ca_4_(PO_4_)_2_O). [1–4]. Calcium phosphates are known to take a particular position among implant materials of various types used in replacement of bone tissue thanks to the fact that during degradation they can be used for formation of new bone tissue [1]. HA may be of synthetic (SHA) or biogenic (BHA) origin, herein the latter is a natural nanostructured material that keeps polyporous micro- and nanostructure of natural bone tissue [5, 6, 20, 21]. Besides, bioactive glass exhibits biological activity as well and is capable to resorb and be replaced with newly formed bone tissue [7–9]; so does bioglass ceramic [17–19].

By today, there have been developed a number of composite materials based on HA and glass (HA/glass), where SHA and BHA as well as other materials on the basis of calcium and phosphor are used as a calcium phosphate component [10–15, 20–22]. All bioactive materials after implantation resorb with a definite rate (depending on the phase composition and structure) followed by replacement of them with newly formed bone tissue. Furthermore, bioceramics characterized by controlled resorption rate can be successfully used as carriers for local drug delivery [7].

In 2010, Marc Bohner described two main approaches used in control of the resorption rate of implant materials for replacement of bone tissue [1]. One is based on the material pore structure modification, in particular on the presence of big enough interconnected pores and channels. Such a porous structure may permit penetration of bone cells, including cell precursors of bone tissue, into blood vessels and thus makes it possible for a material to steadily resorb and so to form new bone tissue. The other approach is based on the chemical composition of material which can modify as well. For instance, over recent years in the field of calcium phosphate materials, interest has shifted from sintered HA to more thermodynamically reactive compounds such as HA/β-tricalcium phosphate (TCP), β-TCP, α-TCP, octocalciumphosphate, dehydrate-dicalcium phosphate, and waterless dicalcium phosphate.

Also, Marc Bohner has indicated that the mechanism of material resorption may be different [1]. Some materials such as HA and β-TCP dissolve under the action of osteoclasts (cells participating in permanent remodeling a human skeleton) and can release some amount of hydrochlonic acid onto the surface of material, which provokes local pH change and calcium phosphate dissolution. Materials of complex composition can resorb through a combination of some mechanisms, which may be inherent for calcium phosphate ceramics or composite ceramics. Among them, namely calcium phosphates, for example HA and β-TCP, are characterized by the presence of self-controlling mechanism during cell resorption of material, which consists in releasing calcium ions and phosphate ions under the action of osteoclasts, which reduces their activity.

To sum up, important properties of a bioactive material are as follows: chemical composition, porosity, and its structure as they significantly affect the behavior of the material under the in vivo conditions. A starting stage of estimation of material bioactivity is in vitro investigation of the material behavior under the conditions of direct contact with a model medium that is an analog of internal medium of organism. As usual, saline and blood serum are used as such model media. There have been developed various procedures for estimation of bioactivity of new materials in these media, starting from studies of change in model medium pH during its contact with a material and material dissolution via weight control during some time.

The aim of the work is to investigate in vitro the behavior of two types of calcium phosphate ceramics on the basis of phosphates of biogenic and synthetic origin with different particle size of initial mixture, which can make it possible to compare the structural and chemical approaches in developing biomaterials.

## Methods

### Sample Preparation

Bioactive glass-ceramic composites based on nanostructured biogenic hydroxyapatite (BHA) or synthetic calcium phosphates mixture (SCPM) with addition of sodium borosilicate glass (mass% 49.10 SiO_2_; 28.14 Na_2_O; 22.76 B_2_O_3_) were prepared as described in [22, 23]. Glass-ceramic composite HA/glass materials were produced using powders of nanostructured BHA (or SCPM) and sodium borosilicate glass with a mass ratio of 0.46:1.0. Powder mixture for SCP/glass composites were produced by mixing sodium borosilicate glass and SCPM (Ca_10_(PO_4_)_6_(OH)_2_, Ca_2_P_2_O_7_, Ca_4_(PO_4_)_2_O and Ca_2_P_2_O_7_) powders (<160 μm). To prepare a BHA/glass mixture, BHA and sodium borosilicate glass were used with two different particle sizes: namely <50 μm (composite BHA/glass-50) and <160 μm (composite BHA/glass). Compact samples of 3 g and 15 mm diameter were fabricated by dry pressing under a pressure of 150 MPa and sintering at 800 °C for all composites studied.

### Characterization Methods

#### Structure

The structure of the composites was studied by scanning electron microscopy (SEM) using a REM-106I (VAT SELMI, Ukraine) microscope and analyzed with the special material science complex program for analysis of structure images SIAMS-600 (SIAMS-Ltd, Russia).

#### Porosity

The composite samples were examined for the apparent density and total and open porosity (*Θ*_t_ and *Θ*_op_).The total porosity of samples (%) was calculated using the formula:

$$ {\varTheta}_{\mathrm{t}}=\left(1-\frac{\rho_{\mathrm{ap}}}{\rho_{\mathrm{pikn}}}\right)\times 100 $$

where *ρ*_ap_ is the apparent density, grams per cubic centimeter;

*ρ*_pikn_ is the pycnometric density of compact material, grams per cubic centimeter.

For BHA *ρ*_pikn_ = 3.00 g/cm^3^.

In order to determine the open porosity, a sample was weighted and saturated with ethylene in vacuum. The saturated samples were weighted in water and in air. The open porosity of samples (%) was calculated by the formula:

$$ {\varTheta}_{\mathrm{op}}=\left({m}_1- m\right)\times {\rho}_{\mathrm{w}}/\left({m}_1-{m}_2\right)\times {\rho}_{\mathrm{liq}} $$

where *m* is the sample weight in air, grams;

*m*_*1*_ is the saturated sample weight in air, grams;

*m*_*2*_ is the saturated sample weight in water, grams;

*ρ*_w_ is the density of water, grams per cubic centimeter;

*ρ*_liq_ is the density of the saturating liquid (ethylene), grams per cubic centimeter.

The closed porosity of samples was calculated by the formula:

$$ {\varTheta}_{\mathrm{cal}} = {\varTheta}_{\mathrm{t}}\hbox{-}\ {\varTheta}_{\mathrm{op}} $$

The structure permeability was determined on the basis of the permeability coefficient which markedly affects the mass transport rate in the course of determination of the dissolution rate in vitro.

$$ k={\varTheta}_{\mathrm{op}}\times {r}^2/8\times \delta $$

where *Θ*_op_ is the open porosity;

*r* is the pore channel radius;

*δ* is the tortuosity of capillary-porous structure.

Based on the data on pore distribution obtained in examination of microstructure of various glass ceramics, average diameters of pore channels of capillary-porous structures were determined. Taking into account the results for pore size distribution, in further calculations, average diameters for the fracture surface were used as most explicit ones.

### In Vitro Bioactivity Testing

Investigation of solubility in vitro of porous samples was carried out in an isotonic saline solution (0.9% NaCl) at a solid/liquid ratio of 1:30 after 2 and 7 days exposition in a thermostat at 36.5 ± 0.5 °C followed by determination of mass loss on an analytic balance “OHAUS Pioneer PA214C” (“OHAUS Corporation,” China) with an accuracy of 0.0001 g.

## Results and Discussion

**Fig. 1:**
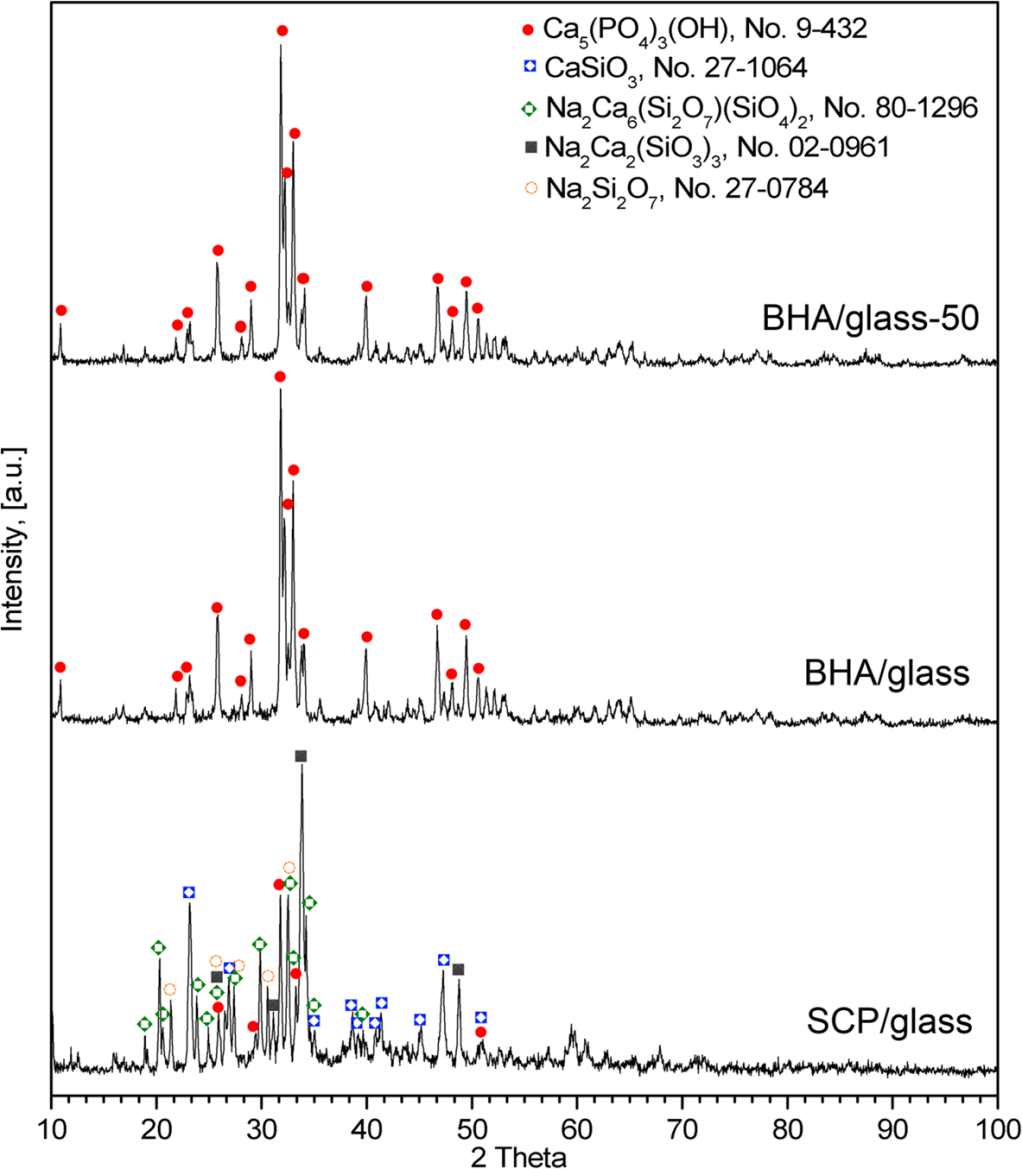
XRD patterns for different types of glass-ceramic composites obtained at 800 °C [22, 23]

The results of the study of the phase composition of composites are shown in Fig. 1. It was established that HA keeps its phase composition in BHA/glass composites. In the case of SCP/glass composites, where mixture of synthetic calcium phosphates was used as a calcium phosphate component, formation of glass ceramics (calcium silicate, sodium calcium silicates, melilite, sodium silicate, sodium borate, HA) took place.

**Fig. 2:**
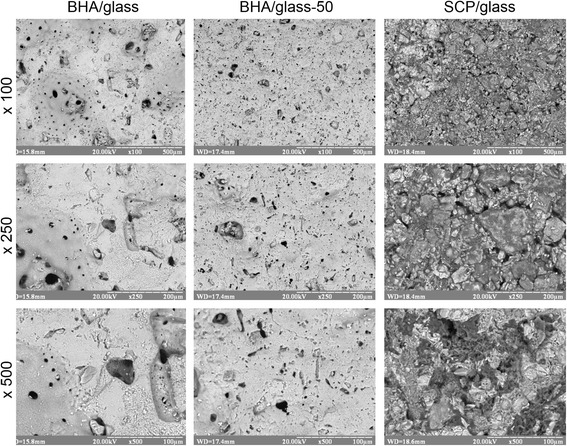
Surface microstructure of different types of glass-ceramic composites

**Fig. 3:**
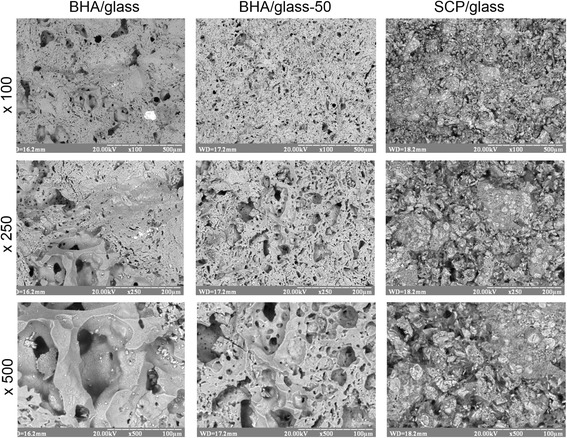
Fracture surface microstructure of different types of glass-ceramic composites

Figures 2 and 3 represent the microstructure of surface and fracture of composites studied. One can see a significant difference in the surface and fracture for all of samples studied, which is clearly seen in BHA/glass composites and somewhat slighter in SCP/glass ones. The fracture microstructure is characterized by more porous structure compared to that of the surface, which may be the result of the surface «vitrification» under sintering, that is, prevailance of glass phase on the surface rather than in the volume. The type of initial phosphate component markedly affects the formation of microstructure and sintering of composites, in particular their grain shape. In the course of phase transformations in bioceramics, needle-like grains are formed sizing within 1.0–19.5 μm, which is characterizing for wollastonite, whereas in BHA/glass composites, individual grains are hardly distinguished due to the material vitrification. In addition, the microstructure of both surface and fracture is characterized by decrease in pore size with decreasing the initial particle size. As a whole, it is evident that the microstructure of the internal part of samples is polyporous, in other words, it is characterized by the presence of multi-sized pores, which is promising in view of the achievement of high resorption properties of bioceramics under direct contact with human organism media.

**Fig. 4:**
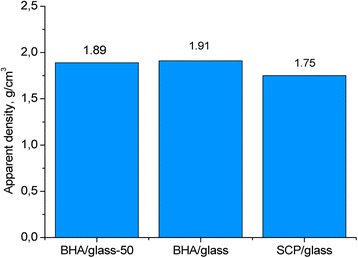
Apparent density of different types of glass-ceramic composites

Figure 4 demonstrates the data on the apparent density of bioceramics obtained. As seen, the apparent density is much affected by both the particle size of initial mixture and the type of calcium phosphate component. The apparent density of the BHA/glass composite is the highest.

**Fig. 5:**
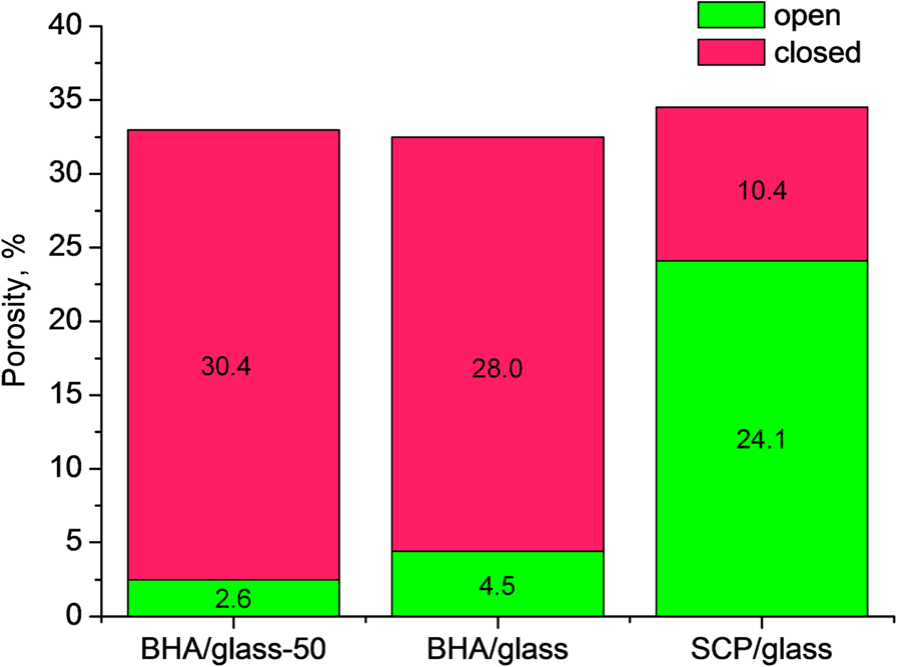
Porosity structure of different types of glass-ceramic composites

The investigation results for the total porosity and its open and closed fractions are presented in Fig. 5. As seen, the total porosity of samples is 32.5–34.5% and practically does not depend on the type of phosphate component and particle size of initial mixture. However, in BHA/glass materials, a closed porosity prevails, whereas in SCP/glass ones—open porosity. The latter can be attributed to running processes of phase formations in synthetic calcium phosphates under sintering accompanied by gas formation, which leads to the obtained porosity structure. As for BHA/glass materials prepared from initial mixtures of different particle size, their porosity falls in a narrow range close to that of SCP/glass materials. The fraction of open porosity in these materials is also much lower and practically does not depend on the particle size of initial mixture.

In determination of solubility in vitro, also of importance is the permeability of porous structure, which depends rather on open porosity, which is effective during material impregnation, and the construction of capillary-porous channels, characterized by high tortuosity, than on the porosity as a whole and the structure of pores. Generally, the permeability of structure is determined by the permeability coefficient, which significantly influences the mass transport rate in determination of the dissolution rate in vitro.

The average diameters of pore channels established through examination of microstructure and pore size distribution were equal to 4.6, 4.7, and 8.83 μm for BHA/glass-50, BHA/glass, and SCP/glass, respectively.

Structure change for materials with the same composition and different initial particle size (BHA/glass) can be estimated mathematically using relations for permeability coefficients:

$$ {k}_1/{k}_2={\varTheta_{\mathrm{op}}}_1\times {\left({d}_{\mathrm{av}1}\right)}^2\times {\delta}_2/{\varTheta_{\mathrm{op}}}_2\times {\left({d}_{\mathrm{av}2}\right)}^2\times {\delta}_1 $$

For limit cases:

(1) at δ_2_/δ_1_ = 1, *k*_1_/*k*_2_ = (0.026 × (4.6)^2^)/(0.045 × (4.7)^2^) = 0.553;

(2) at δ_2_/δ_1_ = 1.5, *k*_1_/*k*_2_ = (0.026 × (4.6)^2^) × 1.5/(0.045 × (4.7)^2^) = 0.829.

The relations give *k*_1_ = 0.553 × *k*_2_ and *k*_1_ = 0.829 × *k*_2_ for (1) and (2) cases, respectively.

For a tortuosity of δ = 1.2–1.6, calculation yields *k*_2_ *=* ﻿(﻿0.045 × (4.7)^2^/4)/(8 × 1.2) = 0.026 μm^2^, herein *k*_1_ for the structure BHA/glass-50 is lower, namely within *k*_1_ = 0.553 × 0.026 = 0.014 μm^2^ (1); *k*_1_ = 0.829 × 0.026 = 0.021 μm^2^ (2).

For the capillary-porous structure of the SCP/glass composite where open porosity dominates (24.1%), at different values of tortuosity, the permeability is equal to *k*_3_ = (0.241 × (8.83)^2^/4)/(8 × 1.2) = 0,489 μm^2^ and *k*_3_ = (0.241 × (8.83)^2^/4)/(8 × 1.6) = 0.367 μm^2^.

As seen, in the case of similar tortuosity, the permeability of the SCP/glass composites is much higher as compared to the other structures studied (*k*_3/_ *k*_2_ = 0.489/0.026 ≈ 19), which is a significant factor, in addition to the composite phase composition, for interpretation of different behavior of porous materials after saturation and solution in vitro.

**Fig. 6:**
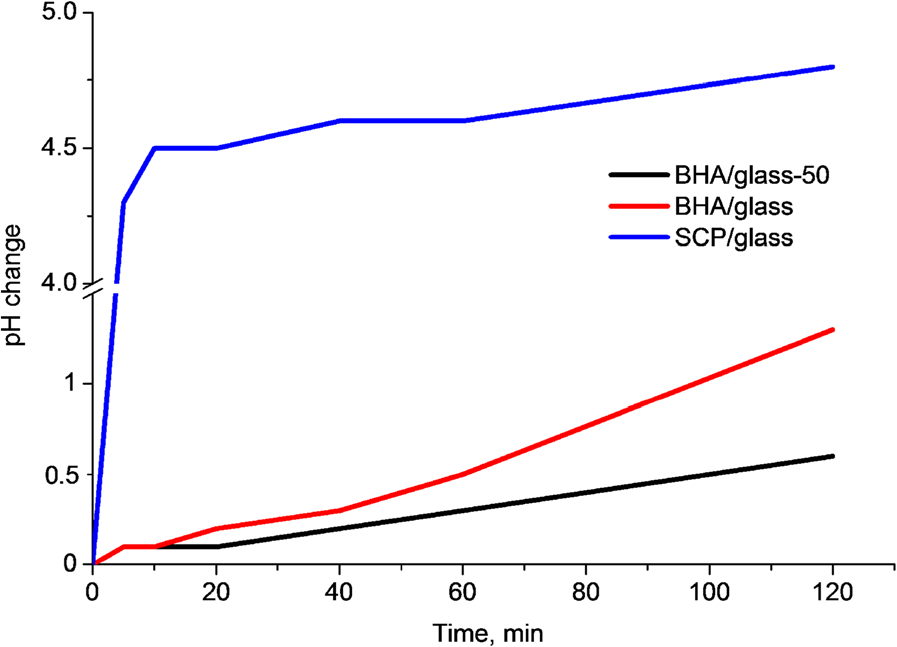
Saline pH change for different types of glass-ceramic composites samples for 120 min sample immersion

Investigation of saline pH change during the first hours upon immersion of composite samples (Fig. 6) showed a sharper change in saline pH after contact with the SCP/glass composite as compared to the BHA/glass composites. Therefore, one may conclude that it is the type of phosphate component that plays an important part in primary estimation of sample solubility in vitro. Herein, comparison of change in saline pH for samples with different initial particle size has revealed that decrease in the particle size very slightly increases saline pH change.

**Fig. 7:**
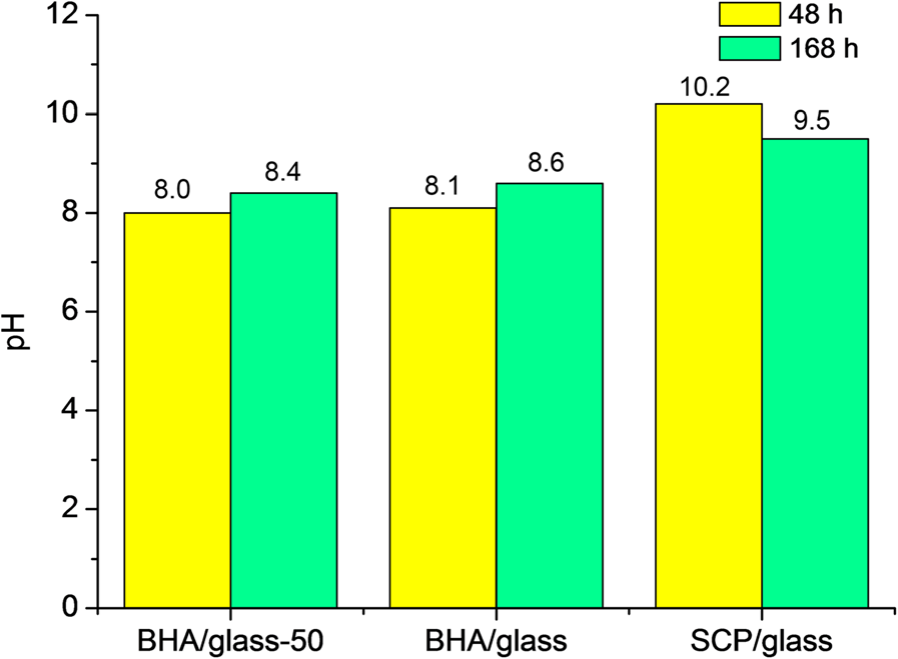
Saline pH after contact with composite samples for different types of glass-ceramic composites (48 and 168 h)

Further changes in saline pH, after 48 h contact with samples, confirmed the observed tendency within the initial 2 h (Fig. 7). The highest pH = 10.2 is achieved by the SCP/glass composite, which was expected yet after 2 h immersion. The BHA/glass composites have much lower pH which is not over 8.1. In addition, such a relation is confirmed: the lower particle size of initial mixture, the more significant pH change. The results obtained after 124 h contact with samples demonstrate change in saline pH depending on the phosphate component used. Also, the BHA/glass composites go on increasing pH up to 8.4–8.6 depending on the initial mixture composition. Unlike in SCP/glass composite, another tendency is observed: pH decreases down to 9.5. A conclusion can therefore be made that for 168 h a maximal pH for SCP/glass is achieved at 48 h observation, whereas for BHA/glass composites this occurred at 168 h.

Generally, concerning changes in saline pH due to contact with composite samples, the following conclusion can be drawn: SCP/glass composites promote more rapid and marked change in saline pH than BHA/glass composites do, which is conditioned, to addition to other important factors, by more complex phase composition, including the presence of various phases of calcium phosphate (calcium silicate, sodium calcium silicates, melilite, sodium silicate, sodium borate, HA), some of which possess higher solubility as compared to BHA. This is consistent with the data of [19], where the solubility orders: glassy phase > CaSiO_3_ > α-Ca_3_(PO_4_)_2_ > β-Ca_3_(PO_4_)_2_, that is the presence of CaSiO_3_ in SCP/glass composite promotes an increase in the dissolution rate. This fact may be responsible for the pH sharp increase in SCP/glass composites after 48 h and its further slight decrease, because high-solubility phases dissolve earlier.

**Fig. 8:**
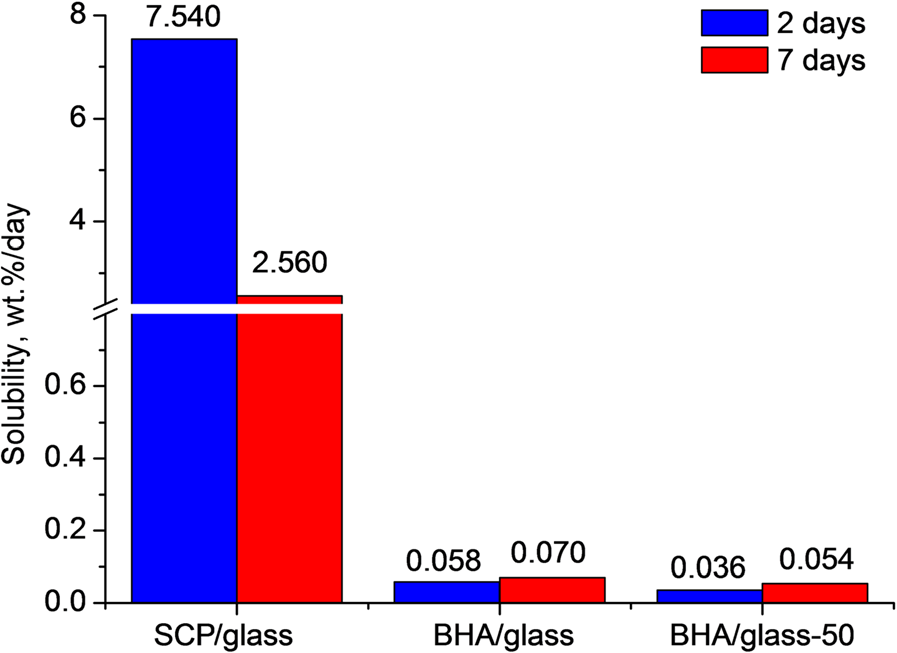
Solibility of glass-ceramic composite samples in saline for 2 and 7 days

Investigation in vitro after 2 and 7 days saline exposition of all of the composites studied revealed that SCP/glass composites have the highest solubility (7.5%mass/day) (Fig. 8). At very close total porosity, solubility of BHA/glass composites is several times lower, which may be related not only to the difference in the phase composition but also to the structure of porosity, in particular to higher fraction of open porosity in SCP/glass composites compared to BHA/glass ones (Fig. 5). The particle size of initial mixture slightly influences the solubility of BHA/glass composites: the solubility of composite with a particle size of initial mixture of <50 μm is some lower than that of composite with a particle size of <160 μm.

Moreover, it was established that at a higher solubility of SCP/glass composites as a whole, the highest solubility was fixed for 2 days saline exposition. After 7 days, this parameter decreased by three times. Unlikely, solubility of BHA/glass composites increased with increasing the time of contact with saline, which can be conditioned by the peculiarities of composite pore structure as well.

## Conclusions

In vitro investigation of two types of calcium phosphate ceramics of biogenic and synthetic origin with different particle size of initial mixture has shown that several different factors influence the behavior of composites during dissolution in vitro. The following factors significantly influence the solubility of composite materials with close composition and different type of calcium phosphate component under the same conditions: phase composition, total porosity, fraction of open porosity, and average diameter of pore channels.

Investigation of composite solubility in vitro after 2 and 7 days contact with saline has established that SCP/glass composites have the highest solubility among materials studied. First of all, this may be related to the peculiarities of their phase composition, high fraction of open porosity, and high permeability. The solubility of BHA/glass composites is several times lower despite the fact that their total porosity is close to that of SCP/glass materials. The particle size of initial mixture practically does not affect the solubility of BHA/glass composites but it is slightly lower for composites with lower particle size (<50 μm). The highest solubility was fixed within 2 days contact with saline, and after 7 days, this parameter decreased by three times. In contrast, solubility of BHA/glass composites increases with increasing the contact time, which may be assigned to the peculiarities of the composite pore structure.

The carried out investigation has demonstrated that the studied composites meet all of the principal requirements for implant materials and are promising in replacement of bone tissue. In particular, SCP/glass materials may be recommended as carriers of drugs when it is necessary to release high doses of drugs, for example antibiotics, during first 2 days after operation. As for BHA/glass materials, they are promising for application as carriers when it is necessary to prolong release of drugs.
